# Electrical Conductivity Response of Poly(Phenylene-vinylene)/Zeolite Composites Exposed to Ammonium Nitrate

**DOI:** 10.3390/s100605590

**Published:** 2010-06-03

**Authors:** Jirarat Kamonsawas, Anuvat Sirivat, Sumonman Niamlang, Pimpa Hormnirun, Walaiporn Prissanaroon-Ouajai

**Affiliations:** 1 Center of Excellence in Petroleum Petrochemicals and Advanced Materials, The Petroleum and Petrochemical College, Chulalongkorn University, Soi Chula 12, Phayathai Road, Phatumwan, Bangkok, 10330, Thailand; E-Mail: som_news@hotmail.com (J.K.); 2 Department of Materials and Metallurgical Engineering, Faculty of Engineering, Rajamangala University of Technology Thanyaburi, Rangsit-Nakhonnayok Road, Klong 6, Thanyaburi, Phatumthani, 12110, Thailand; E-Mail: tanggggo@hotmail.com (S.N.); 3 Department of Chemistry, Faculty of Science, Kasetsart University, 50 Phahonyothin Road, Ladyao, Chatuchak, Bangkok, 10900, Thailand; E-Mail: fscipph@ku.ac.th (P.H.); 4 Department of Industrial Chemistry, Faculty of Applied Science, KMIT-NB, 1518 Pibulsongkram Road, Bangsue, Bangkok, 10800, Thailand; E-Mail: pwalaip@hotmail.com (W.P.-O.)

**Keywords:** conductive polymer, gas sensor, poly(*p*-phenylenevinylene), ammonium nitrate, zeolite Y

## Abstract

Poly(*p*-phenylenevinylene) (PPV) was chemically synthesized via the polymerization of *p*-xylene-bis(tetrahydrothiophenium chloride) monomer and doped with H_2_SO_4_. To improve the electrical conductivity sensitivity of the conductive polymer, Zeolites Y (Si/Al = 5.1, 30, 60, 80) were added into the conductive polymer matrix. All composite samples show definite positive responses towards NH_4_NO_3_. The electrical conductivity sensitivities of the composite sensors increase linearly with increasing Si/Al ratio: with values of 0.201, 1.37, 2.80 and 3.18, respectively. The interactions between NH_4_NO_3_ molecules and the PPV/zeolite composites with respect to the electrical conductivity sensitivity were investigated through the infrared spectroscopy.

## 1. Introduction

The combustion of petroleum products such as diesel oil, heating oil, and heavy fuel oil generates pollutant emissions in the environment. Carbon monoxide (CO) causes chest pain in heart patients, headaches, nausea and reduced mental alertness. Sulfur dioxide (SO_2_) can induce lung disease and breathing problems for asthmatics. Emissions of sulfur dioxide also lead to the deposition of acid rain and other acidic compounds. Such deposition can change the chemical balance of soils which leads to the leaching of trace minerals and nutrients critical to trees and plants. In addition to those toxic gases present in atmosphere, terrorist bomb explosions are of main concern. A safe method is required to detect in advance the potential explosion via tailored gas sensors which can sense various volatiles of typical bomb chemicals: cyclotrimethylenetrinitramine (RDX), trinitrotulene (TNT), and ammonium nitrate [1–4].

Conducting polymers such as poly(*p*-phenylenevinylene) (PPV) can serve as a potential sensing material because PPV possesses good optical and electrical properties, and it can be synthesized by a relative simple technique. To obtain an analyte-specific material, the sensors should have very narrow chemical specificity with high sensitivity towards polar chemicals [5,6]. A zeolite is chosen as a selective microporous adsorbent to be introduced into the polymer matrix in order to increase sensitivity towards NH_4_NO_3_ [7–9]. PPV and their oligomers have been shown to be useful as active sensing materials. PPV has been shown to detect eight organic solvents (chloroform, acetone, ethanol, ethyl acetate, toluene, hexane, acetic acid, methanol, diethyl ether); their sensitivity values were between 10–40% [10]. In our work, we demonstrate further that PPV can detect NH_4_NO_3_ vapor, commonly found in fertilizers and explosive material industries [11]. In this paper, PPV/zeolite Y composites were prepared and electrical conductivity sensitivities of the composites under the NH_4_NO_3_ exposure were investigated, and data were compared with those of pristine PPV and zeoltite Y. Influence of Si/Al ratios of the zeolite Y on the electrical conductivity sensitivities was studied. Based on IR spectroscopy studies, interactions between NH_4_NO_3_ molecules and PPV, zeolite Y, and the PPV/zeolite Y composites are proposed.

## 2. Experimental

### 2.1. Materials

α, α’-Dichloro-*p*-xylene, tetrahydrothiophene and methanol (Aldrich) were used to synthesize *p*-xylene-bis(tetrahydrothiophenium chloride) monomer. Sodium hydroxide (Merck) and hydrochloric acid (Merck) were used as the basic and the acidic reagents, respectively. Sulfuric acid (Merck) was used as the oxidant. Zeolites: CVB400 (Si/Al = 5.1, H^+^), CVB720 (Si/Al = 30, H^+^), CVB760 (Si/Al = 60, H^+^) and CVB780 (Si/Al = 80, H^+^) (all from Zeolyst) were used as the adsorbents. Ammonium hydroxide (Suksapan, Thailand) and nitric acid (Fluka) were used to make ammonium nitrate (NH_4_NO_3_) as the target chemical. Nitrogen (N_2_, TIG) was used as the surface cleaning gas and to vaporize ammonium nitrate. All chemicals were used without further purification.

### 2.2. Poly(p-phenylenevinylene) Synthesis and Doping Process

Synthesis of the *p-*xylene-bis(tetrahydrothiophenium chloride) monomer was achieved by reacting α,α’-dichloro-*p*-xylene with tetrahydrothiophene [12]. The precursor sulfonium polyelectrolyte was prepared in an aqueous solution by the base induced polymerization of an appropriate *bis*-sulfonium monomer. The polymerization reaction was terminated by the addition of dilute aqueous hydrochloric acid to the reaction mixture which was then dialyzed against water in order to separate the high molecular weight fraction from the monomeric and oligomeric residues as well as the sodium and chloride ions. Poly(*p*-phenylenevinylene) (PPV) was obtained by heating pol[(*p*-xylene-bis(tetrahydro-thiophenium chloride)] under vacuum at 180 °C for 6 hours [12]. 18 M sulfuric acid was used as a dopant solution at the mole ratios between PPV repeating unit per sulfuric acid equal to 1:300. The doping process occurred after adding the dopant solution to a polymeric powder, and it was monitored by observing the color changes of the powder from bright yellow to black [13].

### 2.3. Composite Preparation

dPPV/zeolite composites were prepared by dry mixing doped PPV particles with the zeolites at a volume ratio equal to 10:90. The composites were compressed into a disc form by using a hydraulic press at a pressure of 6 kN.

### 2.4. Characterization

FT-IR spectrometry (Bruker, model FRA 106/S) was used to characterize functional groups. A scanning electron microscope (SEM, JEOL, model JSM-5200) was used to study the morphology of PPV, doped PPV, the zeolites, and PPV/zeolite composites at magnifications of 1,500 and 5,000 and at 15 kV. BET (Sorptomatic-1990) was used to measure the pore sizes and the surface areas of the zeolites. A custom made two-point probe with a linear geometric array was used to measure the specific electrical conductivity of samples.

### 2.5. Electrical Conductivity and Gas Measurements

The electrical conductivity of the PPV pellets under exposures to air, N_2_, and NH_4_NO_3_ were measured using a custom made two-point probe which was connected to a voltage supplier (Keithley, 6517A), in which its voltage was varied and the resultant current was measured. The electrical conductivity was calculated by using the equation: *σ = (I/KVt)*, where *I* is the measured current (A), *V* is the applied voltage (V), *t* is the thickness, and *K* is the geometric correction factor of the two-point probe as determined by calibrating the probe with a silicon wafer with a known resistivity value. The electrical conductivity response and sensitivity of the composites were determined by following the equations: Δσ = σ_NH4NO3_ −σ_N2,initial_ and Δσ/σ_N2__,__initial_, respectively. Δσ is the difference in the specific electrical conductivity (S/cm), σ_N2,initial_ is the specific electrical conductivity in N_2_ before exposure (S/cm), and σ_NH4NO3_ is the specific electrical conductivity under NH_4_NO_3_ exposure (S/cm).

## 3. Results and Discussion

### 3.1. Electrical Conductivity Sensitivity of PPV, dPPV and Zeolite Y Exposed to NH_4_NO_3_

The electrical response (Δσ = σ_NH4NO3_−σ_N2initial_ [S/cm]) of each sample was calculated by the difference between the saturated electrical conductivity when exposed to NH_4_NO_3_ and the steady state conductivity value when exposed to pure N_2_ at 1 atm and 30 ± 2 °C. Due to appreciable differences in initial conductivity between various composites, the sensitivity (sensitivity = Δσ/σ_N2_), defined as the electrical conductivity response divided by the electrical conductivity when exposed to pure N_2_, will be used for comparison purposes.

When PPV and dPPV is exposed to NH_4_NO_3_ at 377 ppm, its electrical conductivity increases and the corresponding electrical conductivity sensitivity values are 5.55 × 10^−2^ and 9.65 × 10^−1^, respectively. The positive increment of the sensitivity upon exposed to NH_4_NO_3_ implies that NH_4_NO_3_ molecules act as a primary and secondary dopants for PPV and dPPV, respectively, resulting in a greater number of charges along the polymer backbone.

**Figure 1.**
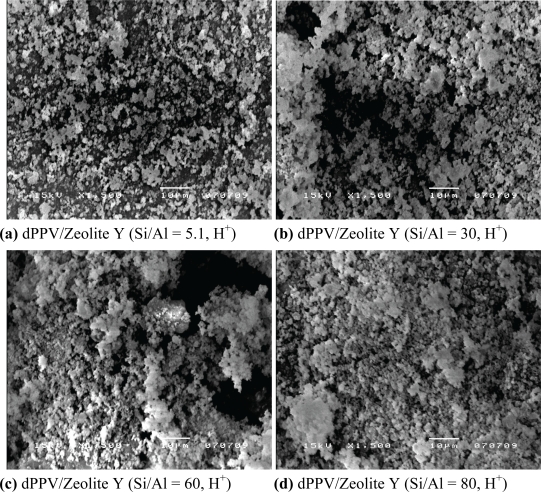
SEM micrographs of the dPPV/zeolite Y composites having different Si/Al ratios (magnification 1,500, 15 kV).

In this study, the zeolites Y having different Si/Al ratios (Si/Al = 5.1, 30, 60, 80, H^+^) were selected to investigate the effect of Si/Al ratios on the electrical conductivity sensitivity towards NH_4_NO_3_. Figures 1(a)–(d) show that the zeolites have nearly the same morphology. In addition, the zeolites Y with different Si/Al ratios possess nearly the same surface areas, pore sizes, and densities. When the zeolites Y are exposed to NH_4_NO_3_ at 377 ppm, the electrical conductivity values increase by one order of magnitude, relative to the values when exposed to nitrogen. The electrical conductivity sensitivity, Δσ/σ_N2_, increases with the increasing Si/Al ratios as shown in Table 1.

A higher Si content of the zeolite Y, as accompanied by a greater amount of cations present, appears to facilitate the static interaction between oxygen on the Si molecule on the zeolite Y and NH_4_NO_3_ [14,15]. The dPPV/zeolite Y composites were fabricated by mixing of the dPPV and zeolite Y having different Si/Al ratios (Si/Al = 5.1, 30, 60, 80, H^+^). All composites contain 90% by volume of the zeolites Y. Zeolite Y particles appear to possess the irregular shape of crystals and appear to be inhomogenously dispersed in the polymer matrix [15].

**Figure 2.**
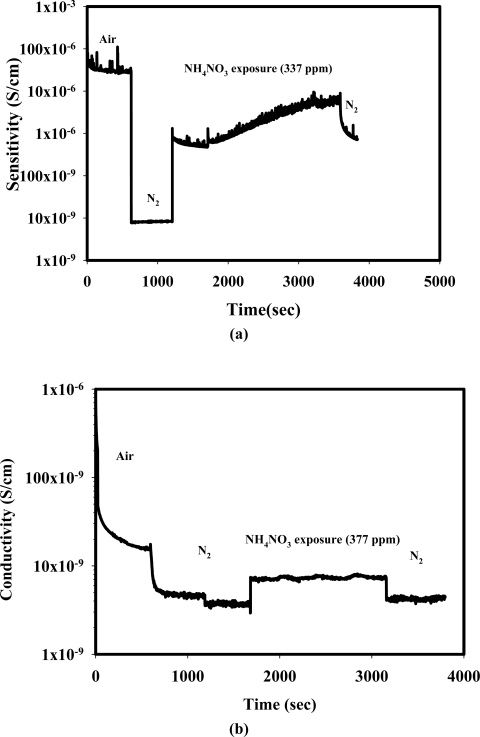

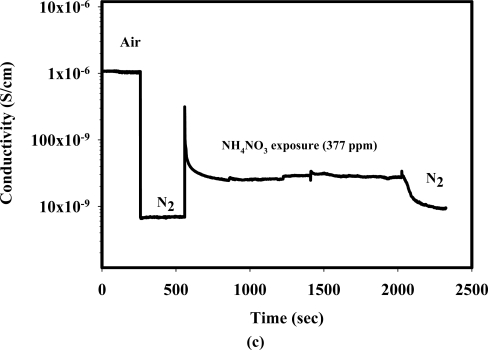
The responses of: (a) doped PPV; (b) Zeolite Y (H^+^, Si/Al = 5.1); and (c) dPPV/Zeolite Y (H^+^, Si/Al = 5.1).

Figures 2(a)–(c) show the response of the doped PPV, Zeolite Y (Si/Al = 5.1, H^+^) and dPPV/zeolite Y (Si/Al = 5.1, H^+^) composite when exposed to NH_4_NO_3_. It is obvious that the doped PPV and the dPPV/zeolite Y composite show comparable increases in electrical conductivity when exposed to NH_4_NO_3_ whereas the zeolite Y exhibits a slight increase in its electrical conductivity. Table 2 tabulates the electrical conductivity sensitivities and the induction times of the doped PPV/zeolite Y composites having different Si/Al ratios. It can be seen from the Table 2 that a higher sensitivity is observed for the composite containing a higher Si/Al ratio. A similar trend is observed for the induction times; a longer induction time is required for the composite with a higher Si/Al ratio. The composite with a higher Si/Al ratio corresponds with the zeolite containing a greater amount of cations which induces a more favorable interaction between NH_4_NO_3_ molecules and the active sites on the conductive polymer chain [16].

**Figure 3.**
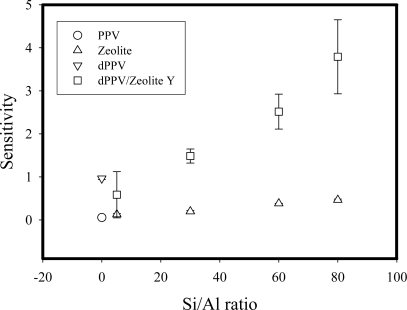
Electrical conductivity sensitivity of PPV, dPPV, Zeolite Y, and dPPV/zeolite Y composites of different Si/Al ratios.

Figure 3 shows that all of the composites have larger sensitivity values than that of the pristine PPV and the doped PPV with the corresponding sensitivity values of 5.55 × 10^−2^, 9.65 × 10^−1^, respectively. The increase in the sensitivity values of PPV/zeolite composites relative to those of the pristine PPV and the doped PPV reflects the fact that NH_4_NO_3_ molecules can adsorb into the zeolites by the electrostatic interaction. Therefore, under this condition, a larger amount of NH_4_NO_3_ molecules are available to interact with dPPV chains.

### 3.2. FTIR Investigations of Reactions of Adsorbed NH_4_NO_3_

FTIR spectra of a dPPV, a zeolite Y and a dPPV/zeolite Y composite were taken. The spectra of samples were collected before, during at 15 minutes interval, and after the NH_4_NO_3_ exposure, in order to study the interaction between the samples and NH_4_NO_3_. The IR spectrum of NH_4_NO_3_ recorded in the 700–3,500 cm^−1^ region (not shown here) exhibits the vibrational stretching frequencies of the free NH_4_^+^ molecules at 3,330, 3,300 cm^−1^ [16,17] and of the free NO_3_^−^ molecules at 1,300 – 1,350 cm^−1^, 815 – 840 cm^−1^ [18].

**Figure 4.**
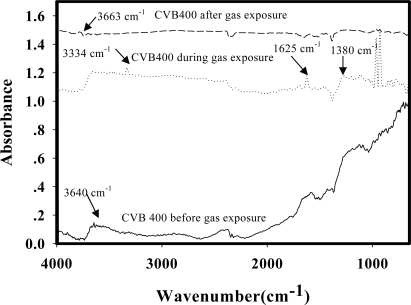
IR spectra of the zeolite Y (Si/Al = 5.1, H^+^) exposed to NH_4_NO_3_ (NH_4_NO_3_ = 377 ppm, pressure at 1 atm, and at T = 25 °C).

Figure 4 shows the IR spectra of the zeolite Y (Si/Al = 5.1, H^+^) before the NH_4_NO_3_ exposure, during the NH_4_NO_3_ exposure, and after the NH_4_NO_3_ exposure. Before the NH_4_NO_3_ exposure, the IR spectrum shows a peak at 3,640 cm^−1^ which can be assigned to the silanol group [19]. During NH_4_NO_3_ exposure, the IR spectrum shows two new peaks at 3,334 and 1,625 cm^−1^; they can be assigned to the interaction between NH_4_^+^ and the oxygen molecules of the zeolite [14,17]. The peak at 1,380 cm^−1^ can be assigned to the interaction between NO_3_^−^ and the oxygen molecules of the zeolite [18]. These three peaks disappears after the NH_4_NO_3_ exposure, and the peak at 3,663 cm^−1^, the stretching vibration of the silanol group, reappears. This suggests that no interaction between the zeolite and NH_4_NO_3_ remains and the interaction is reversible. Overall, there is no significant difference in the zeolite spectra before and after exposure to NH_4_NO_3_ [20–22].

**Figure 5.**
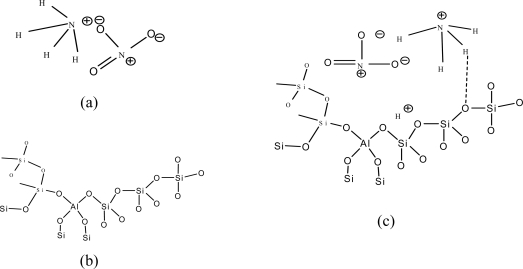
The structures of: (a) NH_4_NO_3_; (b) Zeolite Y structure; and; (c) the interaction between NH_4_NO_3_ and a zeolite.

Figure 5 shows the structure of NH_4_^+^, the zeolite Y structure and the interaction between NH_4_^+^ and the zeolite Y. Zeolite Y (Si/Al = 80) and zeolite Y (Si/Al = 5.1) have comparable specific surface areas: 728 and 868 g/cm^2^. The higher surface area induces more easily the target gas to reside in the cavity [14]. With increasing Si/Al ratio, it appears that the increase in Si in the zeolite Y structure facilitates the static interaction between NH_4_NO_3_ and oxygen on the Si molecule in the zeolite Y. Increasing the static interaction between the target gas and zeolite in turn improves the sensitivity of the PPV/zeolite Y composites as described previously [23,24].

**Figure 6.**
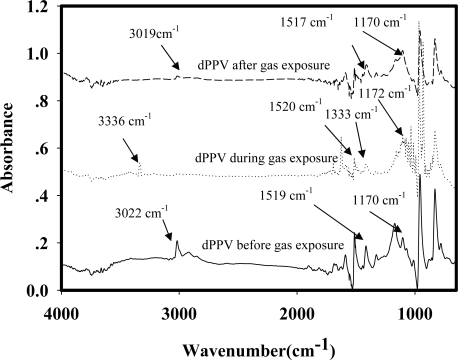
IR spectra of the doped PPV exposed to NH_4_NO_3_ (NH_4_NO_3_ = 377 ppm, pressure at 1 atm, and at T = 25 °C).

**Figure 7.**
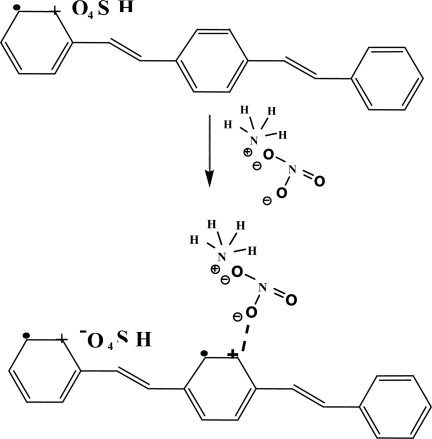
Schematic of the proposed mechanism of the NH_4_NO_3_-dPPV.

Figure 6 shows the IR spectrum of a dPPV before the NH_4_NO_3_ exposure, during the NH_4_NO_3_ exposure, and after the NH_4_NO_3_ exposure. Before the NH_4_NO_3_ exposure, the IR spectrum shows a peak at 1,170 cm^−1^ which can assigned to the quinoid structure, peaks at 1,519 and 3,022 cm^−1^ can be assigned to the phenylene characteristics [10,25]. During the NH_4_NO_3_ exposure, the IR spectrum shows a new peak at 3,336 cm^−1^ which can be assigned to the vibration of NH_4_^+^. The two new peaks at 1,333 and 830 cm^−1^ can be assigned to the vibration of NO_3_^−^ interacting with the cation on the quiniod structure of doped PPV [18]. Increasing peak intensity at 1,172 cm^−1^ during the NH_4_NO_3_ exposure is caused by the increase in the quinoid structures in the doped PPV. The intensities of peaks at 3,019, 1,517 cm^−1^ decrease after the NH_4_NO_3_ exposure and the peaks at 3,336, 1,333 cm^−1^ disappear. The decreases in the intensities at 3,019, 1,517 cm^−1^ after the NH_4_NO_3_ exposure suggest that NH_4_NO_3_ molecules may act as a secondary dopant and the number of the quinoid structures of DPPV increases [18]. From the FTIR result shown in Figure 6, the interaction between the doped PPV and a zeolite Y may be proposed as shown in Figure 7.

**Figure 8.**
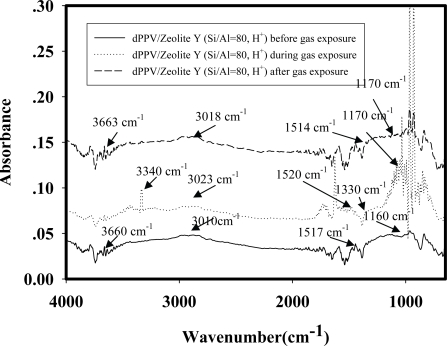
IR spectra of dPPV/zeolite Y (Si/Al = 80, H^+^) exposed to NH_4_NO_3_ (NH_4_NO_3_ = 377 ppm, pressure at 1 atm and at T = 25 °C).

Figure 8 shows the IR spectra of NH_4_NO_3_ (pressure at 1 atm and at room temperature) adsorbed on dPPV/zeolite-Y (Si/Al = 80, H^+^) before the NH_4_NO_3_ exposure, during the NH_4_NO_3_ exposure, and after the NH_4_NO_3_ exposure.

**Figure 9.**
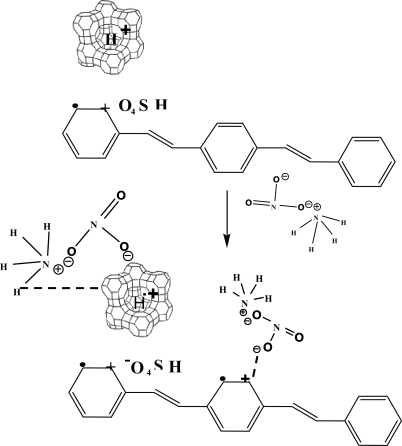
Schematic of the proposed interactions between NH_4_NO_3_ and the dPPV/zeolite Y composite.

Before the NH_4_NO_3_ exposure, the IR spectrum shows a peak at 1,160 cm^−1^ which can be assigned to the quinoid structure, peaks at 1,517 and 3,010 cm^−1^ can be assigned to the phenylene characteristic [10,25], and the peak at 3,660 cm^−1^ can be assigned to the silanol group [19]. During the NH_4_NO_3_ exposure, the IR spectrum shows a new peak at 3,340 cm^−1^ which can be assigned to NH_4_^+^ interacting with oxygen on Si molecule [14,17]. The new peak at 1,330 cm^−1^ can be assigned to NO_3_^−^ interacting with the cation on dPPV and oxygen on Si molecule [18]. The intensities of the peaks at 3,023, 1,520 cm^−1^ decrease during and after the exposure. The decreases in the intensities at 3,023, 1,520 cm^−1^ during and after the exposure suggest that NH_4_NO_3_ molecules may act as a secondary dopant. The number of the quinoid structures increases in the doped PPV structure corresponding to the intensity increase at 1,170 cm^−1^ during the NH_4_NO_3_ exposure. After the NH_4_NO_3_ exposure, the peaks at 3,340, 1,330 cm^−1^ disappear. A peak at 3,663 cm^−1^ is characteristic of the zeolite after the NH_4_NO_3_ exposure, suggesting that no interaction between zeolite and NH_4_NO_3_ remains [17,22]. This is the FTIR evidence for the previously proposed mechanism that zeolite Y induces a larger volume of NH_4_NO_3_ vapor to interact with the doped PPV and NH_4_NO_3_ molecules act as a secondary dopant. A previous study also suggested the interactions between a target gas and a zeolite were further induced by the presence of the zeolite [14]. Figure 9 shows a schematic of the proposed interactions between NH_4_NO_3_ and the dPPV/zeolite Y composites.

## 4. Conclusions

Doped PPV with H_2_SO_4_ is utilized as a NH_4_NO_3_ gas sensing material due to the positive electrical conductivity response. Electrical conductivity sensitivity of the doped PPV towards NH_4_NO_3_ can be improved by introducing the zeolites Y into the doped PPV matrix. The effect of Si/Al ratio was investigated at the ratios of 5.1, 30, 60, and 80. The sensitivity increases monotonically with Si/Al ratio up to 80. The increases in electrical conductivity sensitivity with increasing Si/Al ratio can be described in terms acidity or the amount of cations present on the zeolites. The dPPV/zeolite Y (Si/Al = 80, H^+^) possesses the highest sensitivity of 3.79 since zeolite Y (Si/Al=80, H^+^) has the highest acidity; it can induce a more favorable NH_4_NO_3_ vapor adsorption onto the composite. From FTIR investigations, the NH_4_NO_3_-dPPV interaction is irreversible while NH_4_NO_3_-zeolite interaction is reversible.

## Figures and Tables

**Table 1.** Surface areas, pore size and electrical conductivity sensitivity of the zeolites and the electrical conductivity sensitivity when exposed to NH_4_NO_3_.

**Zeolite**	**BET surface area (m^2^/g)**	**Median Pore width (°A)**	**Apparent Density (g/cm^3^)**	**Sensitivity (Δσ/σ_N2_)**

Zeolite Y (Si/Al = 5.1, H^+^)	864 ± 5.65	10.75 ± 0.0025	2.0046 ± 0.34	1.21 × 10^−1^ ± 7.88 × 10^−3^
Zeolite Y (Si/Al = 30, H^+^)	780 ± 0.35	9.56 ± 0.0982	1.8331 ± 0.27	1.98 × 10^−1^ ± 1.81 × 10^−2^
Zeolite Y (Si/Al = 60, H^+^)	740 ± 28.99	10.74 ± 0.0254	2.0102 ± 0.07	3.83 × 10^−1^ ± 2.55 × 10^−3^
Zeolite Y (Si/Al = 80, H^+^)	728 ± 4.35	10.10 ± 0.0212	2.0048 ± 0.36	4.64 × 10^−1^ ± 2.15 × 10^−2^

**Table 2.** The electrical conductivity sensitivity and the induction and recovery times the dPPV/90%Zeolite Y composites when exposed to NH_4_NO_3_.

**Sample**	**Δσ (σ_NH4NO3_−σ_N2_) (S/cm)**	**Sensitivity (Δσ/σ_N2_)**	**Induction time (minutes)**	**Recovery time, t_r_ (minutes)**

dPPV/90% Zeolite Y (Si/Al = 5.1, H^+^)	2.60 × 10^−3^ ± 3.57 × 10^−3^	5.86 × 10^−1^ ± 5.37 × 10^−1^	41 ± 11	23 ± 8
dPPV/90% Zeolite Y (Si/Al = 30, H^+^)	1.73 × 10^−4^ ± 1.64 × 10^−4^	1.48 × 10^0^ ± 1.64 × 10^−1^	34 ± 11	47 ± 14
dPPV/90% Zeolite Y (Si/Al = 60, H^+^)	1.61 × 10^−3^ ± 2.20 × 10^−3^	2.52 × 10^0^ ± 4.06 × 10^−1^	91 ± 23	38 ± 6
dPPV/90% Zeolite Y (Si/Al = 80, H^+^)	9.73 × 10^−5^ ± 2.02 × 10^−5^	3.79 × 10^0^ ± 8.60 × 10^−1^	118 ± 38	20 ± 10

*σ* = electrical conductivity values, *Δσ* = the electrical conductivity response, and *Δσ/Δσ*_N2_ = electrical conductivity sensitivity, at *T* = 28 ± 1 °C, and at atmospheric pressure.
